# ARF1 promotes prostate tumorigenesis via targeting oncogenic MAPK signaling

**DOI:** 10.18632/oncotarget.9405

**Published:** 2016-05-17

**Authors:** Jason E. Davis, Xiayang Xie, Jianhui Guo, Wei Huang, Wen-Ming Chu, Shuang Huang, Yong Teng, Guangyu Wu

**Affiliations:** ^1^ Department of Pharmacology and Toxicology, Medical College of Georgia, Augusta University, Augusta, GA, USA; ^2^ Cancer Center, Augusta University, Augusta, GA, USA; ^3^ Cancer Biology Program, University of Hawaii Cancer Center, Honolulu, HI, USA; ^4^ Department of Oral Biology, Dental College of Georgia, Augusta University, Augusta, GA, USA

**Keywords:** ARF1, prostate cancer, cell growth, tumorigenesis, Raf1/MEK/ERK1/2

## Abstract

ADP-ribosylation factor 1 (ARF1) is a crucial regulator in vesicle-mediated membrane trafficking and involved in the activation of signaling molecules. However, virtually nothing is known about its function in prostate cancer. Here we have demonstrated that ARF1 expression is significantly elevated in prostate cancer cells and human tissues and that the expression levels of ARF1 correlate with the activation of mitogen-activated protein kinases (MAPK) ERK1/2. Furthermore, we have shown that overexpression and knockdown of ARF1 produce opposing effects on prostate cancer cell proliferation, anchorage-independent growth and tumor growth in mouse xenograft models and that ARF1-mediated cell proliferation can be abolished by the Raf1 inhibitor GW5074 and the MEK inhibitors U0126 and PD98059. Moreover, inhibition of ARF1 activation achieved by mutating Thr48 abolishes ARF1′s abilities to activate the ERK1/2 and to promote cell proliferation. These data demonstrate that the aberrant MAPK signaling in prostate cancer is, at least in part, under the control of ARF1 and that, similar to Ras, ARF1 is a critical regulator in prostate cancer progression. These data also suggest that ARF1 may represent a key molecular target for prostate cancer therapeutics and diagnosis.

## INTRODUCTION

Over the past decades the multiple signaling cascades have been demonstrated to contribute to prostate tumorigenesis [1–7]. Among these pathways, the mitogen-activated protein kinase (MAPK) Raf1/MEK/ERK1/2 pathway, particularly its activation by the cell surface receptor tyrosine kinases (RTKs) and the small GTPase Ras, has been extensively investigated. It is known that the enhanced activation of this MAPK pathway is correlated with the progression, androgen independence and poor prognosis of prostate cancer [8–10] and thus, the molecules involved in the regulation of this pathway has been thought to be the most appealing targets for prostate cancer therapeutics [11, 12]. Despite the fact that multiple naturally occurring mutations have been identified in Ras, Raf1 and MEK which largely contribute to the elevated activation of the MAPK pathway in many other types of cancer [13], these oncogenic mutations are infrequent in prostate cancer patients and the molecular mechanisms underlying the hyperactivation of Raf1/MEK/ERK1/2 pathway in prostate cancer remain poorly defined.

ADP-ribosylation factor 1 (ARF1) is a Ras-related small GTPase and has been well characterized to regulate vesicle-mediated trafficking [14–16]. Like other GTPases, the function of ARF1 is highly regulated by its recycling between active GTP-bound and inactive GDP-bound conformations. The GDP-bound ARF1 may be recruited from the cytosol onto the membrane by interacting with receptor proteins. This process is important for the activation of ARF1 by guanine nucleotide exchange factors [17]. Active GTP-bound ARF1 subsequently interacts with downstream effectors to form transport vesicles. In addition to its well-established trafficking function, ARF1 may also function as a signal transducer to directly activate signaling molecules [18, 19]. We have recently found that ARF1 regulates the activation of the Raf1/MEK/ERK1/2 pathway by α_2B_-adrenergic receptor, a prototypic G protein-coupled receptor (GPCR) [20]. In cancer biology, ARF1 has been shown to regulate proliferation and migration of breast cancer cells which is mediated through the phosphoinositide 3-kinase (PI3K) pathway [21], the retinoblastoma protein [22] and Rac1 [23]. Interestingly, AMF-26, a small molecule octahydronaphthalene derivative, inhibits ARF1 activation and induces complete regression of human breast cancer BSY-1 xenografts [24]. In addition, ARF1 was shown to interact with phosphatase of regenerating liver 3 (PRL-3), a newly identified target in colorectal cancer, and regulate PRL-3-dependent cell migration [25]. However, virtually nothing is known about the function of ARF1 in prostate cancer.

In the current study, we have shown that ARF1 is significantly upregulated in prostate cancer and that manipulation of ARF1 expression is able to control ERK1/2 activation and prostate cancer cell growth. These data have demonstrated for the first time that, by virtue of its ability to activate oncogenic MAPK pathway, ARF1 plays a crucial role in prostate tumorigenesis and thus, may represent a novel therapeutic target for prostate cancer.

## RESULTS

### ARF1 expression is upregulated in human prostate cancer tissues and cells

**Figure 1:**
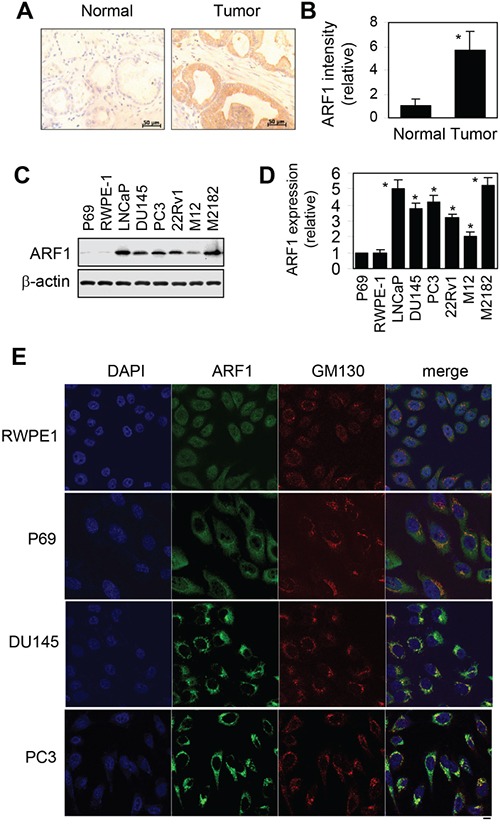
Elevated expression of ARF1 in prostate cancer cells and tumor tissues **A.** analysis of ARF1 expression in prostate tumor tissues by immunohistochemistry. Scale bars, 50 μm. **B.** the relative intensity of ARF1 antibody staining in clinic prostate tissues and tissue microarrays containing 21 normal and 86 prostate tumor samples. *P < 0.05 versus normal tissues. **C.** a representative blot showing ARF1 expression in a variety of prostate cancer cell lines and prostate normal cell lines. The expression of β-actin was used as a loading control. **D.** quantitative data of ARF1 expression shown in C (n=3). *P < 0.05 versus P69 cells. **E.** subcellular distribution of endogenous ARF1 and its colocalization with the Golgi marker GM130 in RWPE1, P69, DU145 and PC3 cells revealed by confocal fluorescence microscopy. The data shown are representative images of at least 3 independent experiments. Scale bar, 10 μm.

As an initial approach to study the possible function of ARF1 in prostate cancer, we studied the expression of ARF1 in human prostate cancer tissues by immunohistochemistry. ARF1 antibody staining of clinic primary prostate tissues and tissue microarray demonstrated that the levels of ARF1 were significantly increased in prostate cancer tissues compared with normal prostate epithelium (Figure 1A and 1B). Oncomine (www.oncomine.org) analysis also showed that the majority of the prostate cancer patients evaluated had elevated expression levels of ARF1 being about 3.5- and 2.5-fold higher in prostate adenocarcinoma and carcinoma, respectively, than in normal prostate gland (Data not shown).

We next compared the expression levels of ARF1 in human prostate normal and cancer cell lines. Similar to human prostate cancer tissues, ARF1 expression was also significantly increased in all tested prostate cancer cell lines, including LNCaP, DU145, PC3, 22Rv1, M12 and M2182, as compared to that in the normal prostate P69 and RWPE1 cells (Figure 1C and 1D). These data demonstrate that ARF1 is highly expressed in prostate cancer.

Confocal microscopy was then used to analyze the subcellular distribution of ARF1 in prostate normal and cancer cells. The results showed that, consistent with the immunoblotting data, ARF1 antibody staining signal was much stronger in DU145 and PC3 cells then in P69 and RWPE1 cells. Interestingly, ARF1 was mainly expressed in the cytoplasm in both P69 and RWPE1 cell lines, whereas it was strongly colocalized with GM130, a Golgi marker, in prostate cancer DU145 and PC3 cells (Figure 1E). These observations suggest Golgi localization of ARF1 in prostate cancer cells. As the activated ARF1 localizes to the Golgi [26–28], these data suggest that ARF1 is more activated in DU145 and PC3 cells than that in P69 and RWPE1 cells.

### ARF1 regulates the activation of ERK1/2 in prostate cancer cells

**Figure 2:**
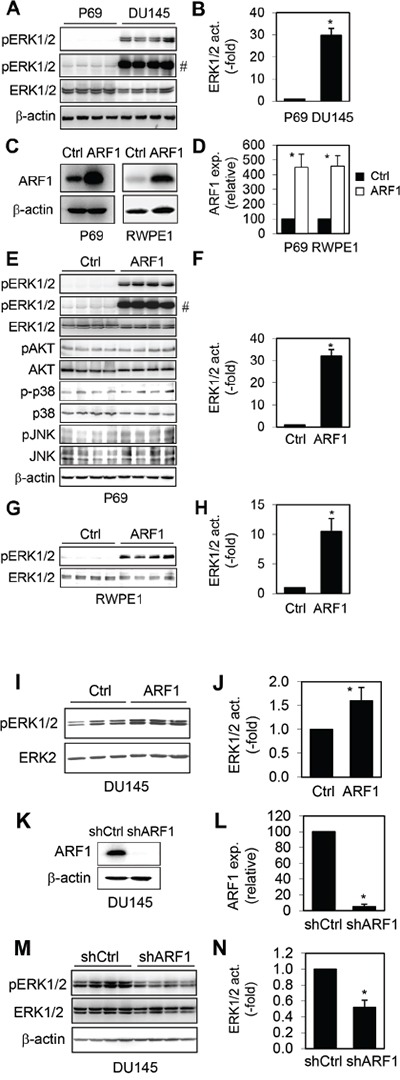
Effect of manipulating ARF1 expression on the activation of ERK1/2 in prostate cancer cells **A.** ERK1/2 activation in P69 and DU145 cells. **B.** Quantitative data of ERK1/2 activation shown in A. **C.** Lentiviral expression of ARF1 in P69 and RWPE1 cells. **D.** Quantitative data of ARF1 overexpression shown in C. **E.** The activation of ERK1/2, AKT, p38 and JNK in P69 cells infected with control lentiviral vector and ARF1. **F.** Quantitative data of ERK1/2 activation shown in E. **G.** The activation of ERK1/2 in RWPE1 cells infected with control lentiviral vector and ARF1. **H.** Quantitative data of ERK1/2 activation shown in G. **I.** The effect of ARF1 expression on the activation of ERK1/2 in DU145 cells. **J.** Quantitative data shown in I. **K.** shRNA-mediated knockdown of ARF1 in DU145 cells. **L.** Quantitative data shown in K. **M.** ERK1/2 activation in DU145 cells infected with control or ARF1 shRNA. **N.** Quantitative data of ERK1/2 activation shown in **M.** # in A and E indicates the blots with longer exposure to show ERK activation in control cells. *P < 0.05 versus P69 cells in panel B and versus respective control in panels D, F, H, J, L and N (n=3-5).

The MAPK ERK1/2 have been well described to be highly activated in prostate cancer [8–10]. Consistent with these reports, we found that ERK1/2 activation was dramatically higher by approximately 28 folds in DU145 cells than that in P69 cells (Figure 2A and 2B).

To investigate if increased ARF1 expression was directly linked to ERK1/2 activation in prostate cancer, we determined if manipulating ARF1 expression could alter ERK1/2 activation. We first determined the effect of lentiviral-mediated expression of ARF1 (Figure 2C and 2D) on the activation of ERK1/2 in normal prostate P69 and RWPE1 cells which have low ARF1 expression. ARF1 overexpression remarkably enhanced ERK1/2 activation by about 32 folds in P69 cells (Figure 2E and 2F). In contrast, ARF1 overexpression did not alter the activation of AKT, p38 and JNK in P69 cells (Figure 2E), suggesting that ARF1 is able to specifically activate the MAPK ERK1/2. Lentiviral expression of ARF1 also dramatically augmented ERK1/2 activation by approximately 10 folds in RWPE1 (Figure 2G and 2H). In addition, enhanced ARF1 expression further increased the activation of ERK1/2 in DU145 cells in which ERK1/2 were highly activated (Figure 2I and 2J).

We then determined the effect of shRNA-mediated knockdown of ARF1 on the activation of ERK1/2 in prostate cancer cells in which ARF1 is highly expressed. For this purpose, we created stable DU145 cells carrying shRNA targeting ARF1 (Figure 2K and 2L). Expression of ARF1 shRNA reduced ERK1/2 activation by 50% in DU145 cells (Figure 2M and 2N). These data indicate that the expression level of ARF1 is an important factor contributing to the enhanced activation of the MAPK ERK1/2 in prostate cancer cells.

### ARF1 regulates prostate cancer cell proliferation and colony formation

**Figure 3:**
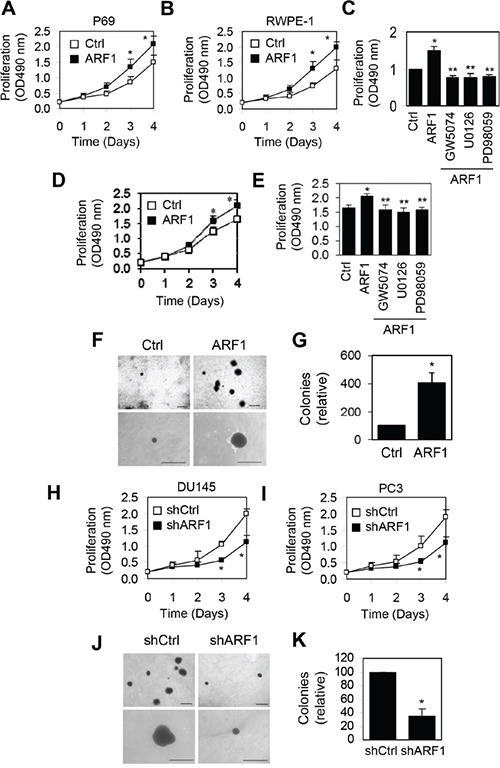
Effect of manipulating ARF1 expression on prostate cancer cell proliferation and anchorage-independent growth **A**, **B** and **D.** effect of lentivirus-mediated overexpression of ARF1 on P69 (A), RWPE1 (B) and DU145 (D) cell proliferation measured by CellTiter 96^®^ AQueous One Solution Cell Proliferation assays. **C** and **E.** effect of treatment with GW5074, U0126 and PD98059 on ARF1-induced P69 (C) and DU145 (E) cell proliferation. **F.** effect of ARF1 overexpression on the colony size and number in P69 cells measured in soft agar assays. **G.** quantitative data shown in **F. H** and **I.** effect of shRNA-mediated ARF1 knockdown on DU145 **(H)** and PC3 **(I)** cell proliferation. **J.** effect of ARF1 knockdown on the colony size and number in DU145 cells. **K.** quantitative data shown in **J.** The data shown in F and J are representative images of at least three independent experiments. Scale bars, 250 μm. *P < 0.05 versus respective control. **P < 0.05 versus ARF1 (n=3-6).

To directly investigate the role of ARF1 in prostate cancer malignancy, we first determined the short-term effect of increased ARF1 expression on cell proliferation. Overexpression of ARF1 in P69 (Figure 3A) and RWPE1 cells (Figure 3B) significantly increased cell proliferation at days 3 and 4. To define if the influence of increased expression of ARF1 on the phenotypic change of P69 cells was directly associated with the activation of the MAPK pathway, we determined the effect of pharmacologically inhibiting this pathway on ARF1-mediated cell proliferation. ARF1-induced cell proliferation was abolished by treatment with the Raf1 inhibitor GW5074 and the MEK inhibitors U0126 and PD98059 (Figure 3C). In addition, lentiviral expression of ARF1 moderately augmented DU145 cell proliferation and this effect was completely blocked by treatment with GW5074, U0126 and PD98056 (Figure 3D and 3E). These data suggest that the effect of ARF1 on cell proliferation is likely induced by activating the Raf1/MEK/ERK1/2 pathway. To examine the long-term effect of ARF1 overexpression on anchorage-independent growth, soft agar clonogenic assay was carried out. ARF1 overexpression markedly increased colony number and size of P69 cells (Figure 3F and 3G).

We then determined the effect of reducing ARF1 expression on the growth of prostate cancer DU145 and PC3 cells. shRNA-mediated knockdown of ARF1 significantly slowed down cell proliferation in DU145 (Figure 3H) and PC3 cells (Figure 3I). Furthermore, soft agar clonogenic assay showed that colony size and number were markedly decreased in DU145 cells infected with ARF1 shRNA compared with the control cells (Figure 3J and 3K). These data indicate that knockdown of ARF1 inhibits prostate cancer cell proliferation and colony formation.

### ARF1 knockdown suppresses prostate tumorigenesis *in vivo*

**Figure 4:**
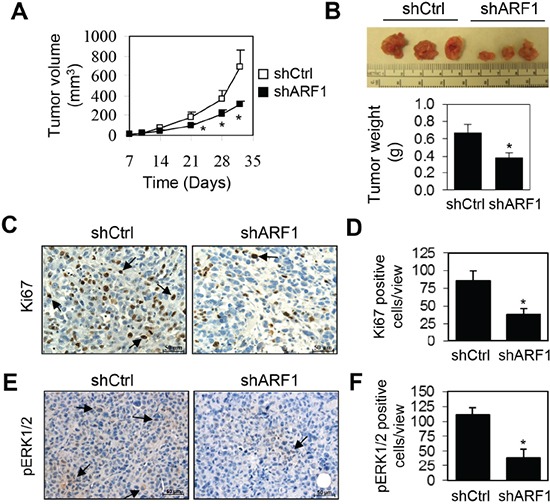
Depletion of ARF1 suppresses prostate tumorigenesis in the mouse-xenograft models **A.** effect of ARF1 knockdown by shRNA on tumor growth in DU145 xenograft models. NOD/SCID mice (n = 10) were injected s.c. with 1.5 × 10^6^ DU145 cells expressing ARF1 shRNA or control shRNA. Individual tumor volumes (mm^3^) were measured at indicated days after implantation. **B.** representative xenografts (n = 10) generated as in A (upper panel) and tumor weight (g) measured at 32 days after implantation (lower panel). **C.** representative images showing the effect of ARF1 knockdown on cell proliferation in xenograft tumors measured by Ki67 antibody staining. Arrows indicate Ki67-positive cells. **D.** quantification of the Ki67-positive cells shown in C. **E.** representative images showing the effect of ARF1 knockdown on the activation of ERK1/2 in xenograft tumors derived from ARF1 knockdown and control cells as measured by pERK1/2 antibody staining. Arrows indicate pERK1/2-positive cells. **F.** quantitative data shown in E. *P < 0.05 versus control. Scale bars, 50 μm.

We further examined the inhibitory effect of ARF1 knockdown on tumor growth in the mouse xenograft model. For this purpose, DU145 cells infected with either control shRNA or ARF1 shRNA were injected subcutaneously into the right flank of NOD/SCID mice (n = 10). DU145 cell-derived tumor growth was partially blocked by the knockdown of ARF1, resulting in an approximately 50% reduction in both tumor volume and weight (Figure 4A and 4B). To further define the role of ARF1 in prostate tumorigenesis *in vivo*, we measured cell proliferation in DU145 xenografts by Ki67 antibody staining. The number of Ki67-positive cells was markedly reduced in the tumors derived from ARF1 knockdown cells as compared with the tumors derived from the control cells (Figure 4C and 4D), indicating that ARF1 affects prostate cancer cell proliferation *in vivo*. We also determined ERK1/2 activation by immunohistochemistry and found that knockdown of ARF1 significantly reduced the number of pERK1/2-positive cells in DU145 xenografts (Figure 4E and 4F). These observations indicate that ARF1 plays an important role in controlling prostate tumorigenesis which is likely mediated through regulating the activation of the MAPK ERK1/2 pathway.

### Inactivation of ARF1 by mutating Thr48 inhibits the function of ARF1

**Figure 5:**
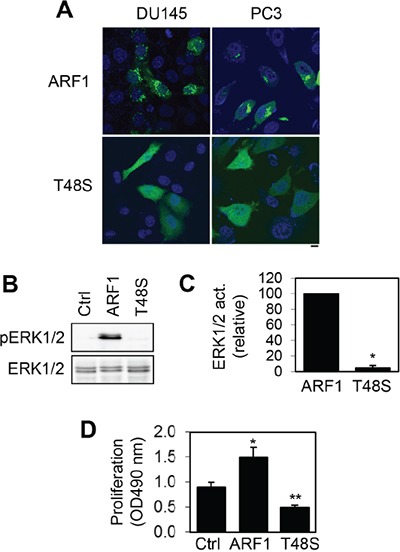
Effect of mutating Thr48 on ARF1 localization and its ability to activate ERK1/2 and promote cell proliferation **A.** subcellular localization of GFP-tagged ARF1 and its mutant T48S in DU145 and PC3 cells revealed by confocal microscopy. The data shown are representative images of at least 5 independent experiments. Scale bar, 10 μm. **B.** effect of transient expression of ARF1 and its mutant T48S on the activation of ERK1/2 in P69 cells. **C.** quantitative data shown in B (n=3). *P < 0.05 versus ARF1. **D.** effect of ARF1 and its mutant ARF1T48S on the proliferation of P69 cells (n=4). *P < 0.05 versus control; **P <0.05 versus both control and ARF1.

To further study the function of ARF1 in prostate cancer cells, we determined the effect of mutating Thr48 on the activation of the MAPK pathway and cell proliferation. Thr48 is a highly conserved residue in switch I region of small GTPases and heterotrimeric G proteins which plays a crucial role in their activation [29–33]. We found that mutation of Thr48 to Ser impaired the Golgi localization of ARF1 in prostate cancer DU145 and PC3 cells and the mutant ARF1T48S was largely expressed in the cytoplasm (Figure 5A).

We then compared the effects of lentiviral-mediated expression of ARF1 and its inactive mutant ARF1T48S on ERK1/2 activation and cell proliferation in P69 cells. Consistent with our previous results, ARF1 overexpression markedly enhanced ERK1/2 activation (Figure 5B and 5C) and cell proliferation in P69 cells (Figure 5D). In contrast, the expression of ARF1T48S did not activate ERK1/2 as compared to control cells (Figure 5B and 5C). Interestingly, cell proliferation was significantly lower in P69 cells expressing the mutant ARF1T48S than that in cells expressing ARF1 or control vector (Figure 5D). These data demonstrate that mutation of Thr48 to block ARF1 activation abolishes its abilities to activate the MAPK pathway and promote cell growth. These data also suggest that ARF1T48S may function as a dominant-negative mutant to inhibit prostate cell proliferation.

## DISCUSSION

There are two important findings presented in this manuscript. One is that we have identified a previously unappreciated function for ARF1 as a crucial mediator of prostate tumorigenesis. We have demonstrated that ARF1 expression is elevated in human prostate cancer tissues and cells and the expression level of ARF1 is directly correlated with prostate tumorigenesis. The other is that we have revealed a novel molecular mechanism underlying the hyperactivation of the Raf1/MEK/ERK1/2 pathway in prostate cancer. Based on these findings, we postulate that, in prostate cancer, elevated expression/activation of ARF1 promotes the activation of the Raf1/MEK/ERK1/2 pathway. As one of the best characterized signaling pathways involved in the progression of prostate cancer, the Raf1/MEK/ERK1/2 pathway is under control by the small GTPase ARF1 which is also upregulated. Therefore, similar to the oncogene Ras and the Ras-mediated MAPK pathway, ARF1 and ARF1-mediated ERK1/2 activation may represent novel molecular targets for prostate cancer therapeutics and diagnosis.

The function of ARF1 in prostate cancer has not been investigated. Our recent studies demonstrating that ARF1 is involved in the activation of the MAPK ERK1/2 pathway which has been implicated in the progression of prostate cancer prompt us to define the possible function of ARF1 in prostate cancer. Indeed, ARF1 was found to be highly expressed in several invasive prostate cancer cells and prostate cancer patients. We then used two different strategies to determine if altered expression of ARF1 correlated with ERK1/2 activation in prostate cancer cells. The first strategy was to define if enhancing ARF1 expression could increase ERK1/2 activation in normal prostate cells and the second strategy was to determine if reducing ARF1 expression could decrease ERK1/2 activation in prostate cancer cells. Consistent with the role of ARF1 in regulating ERK1/2 activation, lentiviral expression of ARF1 in normal prostate epithelial cells dramatically enhanced, whereas ARF1 depletion in prostate cancer cells inhibited ERK1/2 activation. These data strongly suggest that upregulation of ARF1 may contribute to the enhanced activation of ERK1/2 in prostate cancer cells. It has been shown that many MAPK pathway inhibitors, such as the Sprouty family members, the tumor suppressor phosphatase and tensin homolog (PTEN) and Raf kinase inhibitor protein (RKIP) [34–41], are downregulated in prostate cancer cells. Therefore, both downregulation of inhibitors and upregulation of activators contribute to the hyperactivation of the MAPK pathway in prostate cancer.

The same strategies were used to determine if altered expression of ARF1 correlated with prostate cancer cell growth. We demonstrated that, in parallel with the effects on ERK1/2 activation, ARF1 overexpression in normal prostate cells significantly augmented, whereas ARF1 knockdown in prostate cancer cells markedly attenuated cell growth both in cultured cells *in vitro* and in the mouse xenograft model *in vivo*. These opposing effects produced by increasing and decreasing ARF1 expression have revealed for the first time an important function of ARF1-mediated signaling in the tumorigenesis of prostate cancer, implying that suppressing ARF1 expression/function may provide a novel mean for prostate cancer therapeutic.

The function of ARF1 in regulating prostate cancer cell growth is likely mediated through activating the Raf1/MEK/ERK1/2 pathway. This possibility is supported by the fact that ARF1-mediated cell growth was completely blocked by treatments with GW5074, U0126 and PD98059 in both P69 and DU145 cells. Furthermore, in contrast to wild-type ARF1, its mutant T48S was unable to activate the MAPK pathway and inhibited cell proliferation, suggesting it may function as a dominant-negative mutant to block the function of endogenous ARF1 leading to inhibition of cell growth. Moreover, we have shown that ARF1 overexpression did not influence the activation of AKT, p38 and JNK. These data suggest a specific ARF1-mediated pathway contributing to the activation of the Raf1/MEK/ERK1/2 pathway in prostate cancer and that manipulation of ARF1 expression unlikely alters the activation of the PI3K-AKT pathway as defined in breast cancer cells [21]. As ARF1 has been shown to be activated by many cell surface GPCRs and RTKs [20, 21, 42–44], this novel mechanism of the MAPK ERK1/2 activation may be commonly used by these receptors to regulate prostate tumorigenesis.

Multiple naturally occurring mutations have been identified to lead to aberrant MAPK signaling and malignant cell proliferation in many different cancers and these mutated proteins are the actual targets for the treatment. For example, two Raf inhibitors (vemurafenib and dabrafenib) have been recently approved by FDA for the treatment of metastatic melanoma patients carrying a specific mutation of Raf. Unfortunately, these inhibitors cannot be used to treat cancers that lack mutations in Raf [45]. Although hyperactivation of MAPK significantly contributes to the prostate cancer progression, the abovementioned oncogenic mutations are not often observed and the molecular mechanisms underlying the activation of MAPK pathway remain largely undefined in prostate cancer. Our data provide direct evidence implicating that ARF1-mediated Raf1/MEK/ERK1/2 signaling is critical for prostate tumorigenesis and that genetic approaches targeting ARF1 in animal models may provide an important and novel avenue for studying tumorigenesis and developing therapeutics.

## MATERIALS AND METHODS

### Molecular reagents and constructs

ARF1 antibodies were purchased from Assay Designs. Antibodies against phospho-ERK1/2 were from Santa Cruz Biotechnology, Inc. Antibodies against ERK1/2 and GM130 were from Cell Signaling Technology. GW5074 was from Sigma. U0126 and PD98059 were purchased from Calbiochem. ARF1 and its mutant ARF1T48S conjugated with GFP at their C-termini were generated by using the BamHI and EcoRI restriction sites of the pEGFP-N1 vector as described previously [33]. For generation of shRNA in the pLKO.1–TCR vector, two oligos targeting ARF1 were synthesized, annealed and cloned into the EcoRI and AgeI restriction sites of the lentiviral pLKO.1 puro vector (Sigma). To generate ARF1 in the pCDH-CMV-MCS-EF1-copGFP cloning and expression vector (System BioSciences, Mountain View, CA), ARF1 was amplified by PCR and inserted into the EcoRI and BamHI restriction sites. The mutations were carried out using the QuikChange site-directed mutagenesis kit (Stratagene, La Jolla, CA).

### Cell culture, transfection and infection

RWPE1 and prostate cancer cell lines DU145, PC-3, LNCaP, 22Rv1, M12 and M2182 were purchased from American Type Culture Collection (ATCC, Rockville, MD). P69 cell line was generously provided by Dr. Wanguo Liu (Louisiana State University Health Sciences Center, New Orleans, LA). The cells were cultured as described previously [1, 46]. Transient transfection of the cells was carried out using Lipofectamine 2000 (Invitrogen). The lentiviruses driving the expression of ARF1 or shRNA targeting ARF1 were packaged, purified, and applied to the cells according to the manufacture's instruction.

### Measurement of ERK1/2, AKT, p38 and JNK activation

The cells were cultured on 6-well dishes in medium containing 10% fetal bovine serum and transfected or infected with the plasmid expressing ARF1 or ARF1 shRNA. The activation of ERK1/2, AKT, p38 and JNK was determined by immunoblotting measuring their phosphorylation with phosphor-specific antibodies as described previously [20, 33].

### Confocal fluorescence microscopy

Cells grown on coverslips pre-coated with poly-L-lysine in 6-well plates were fixed with 4% paraformaldehyde-4% sucrose mixture in PBS for 15 min, permeabilized with PBS containing 0.2% Triton X-100 for 5 min, and blocked with 5% FBS for 1 h. The cells were then incubated with antibodies against GM130, a Golgi marker, or ARF1 at a dilution of 1:500 for 1 h. After washing (3 × 5 min), the cells were incubated with Alexa Fluor 488- and 594-labeled secondary antibodies (1:1000 dilution) for 1 h. The coverslips were mounted with ProLong^®^Gold antifade reagent (Invitrogen) and images were captured using a LSM510 Zessis confocal microscope equipped with a 63× objective (NA=1.3) as described previously [47, 48].

### Cell proliferation assay

Cells were seeded into 96-well culture plates at a density of 1×10^3^ cells in a final volume of 100 μl/well. The cell proliferation rate was analyzed at different time points (1-4 days) with CellTiter 96^®^ AQueous One Solution Cell Proliferation assay (Promega). For the assay, 20 μl of CellTiter 96^®^ AQueous One Solution Reagent were added to each well followed by incubation for 2 h at 37°C. The absorbance at 490 nm was measured with a microplate reader (Bio-Rad). The average absorbance values from six wells per group were calculated.

### Soft agar colony formation assay

Soft agar colony formation assay was performed using a two-layer soft agar system as described previously [46]. Cells were seeded in 6-well plates at a density of 1×10^4^ cells per well. The cells suspended in 1.5 ml of top agar (0.3% agar in 2×RPMI complete cell culture medium) were overlaid onto a layer of 1.5 ml of bottom agar (0.6% agar in the same culture medium). After 14 days incubation, the colonies were photographed and scored by inverted phase-contrast microscopy without fixation and staining.

### Tumor implantation

All animal procedures were approved by the Institutional Animal Care and Use Committee (IACUC) of Augusta University. Six-week-old male NOD/SCID mice (Jackson Laboratories, Bar Harbor, ME, USA) were used for tumor implantation as described previously [46]. Briefly, 1.5 × 10^6^ DU145 cells infected with control or ARF1 shRNA were resuspended in 100 ml of Matrigel (BD Biosciences, Franklin Lakes, NJ) and subcutaneously injected into the mice. The tumor size was measured externally every 4 to 7 days by a caliper as length × width^2^/2. The mice were euthanized on day 32 post-injection by CO_2_ inhalation, and tumors were dissected out and individually weighed.

### Immunohistochemistry

The human primary prostate cancer tissues were obtained from Augusta University tumor bank with institutional review board approval. The prostate tissue microarray was purchased from US Biomax (Rockville, MD). Immunohistochemistry of the human tissues and tissue microarray were conducted as described previously [46, 49] using ARF1 antibodies (1:500). For quantifying staining intensity, 10 random microscopic fields were captured by a CCD camera (Olympus, Center Valley, PA) and signal intensity was determined using the Image-Pro Plus software (MediaCybernetics, Rockville, MD). In tumor implantation experiment, the mice were sacrificed on day 32, and the tumors were removed and processed for immunohistochemistry. Ki67 (1:500) and pERK1/2 (1:250) antibodies were used to examine cell proliferation and ERK1/2 activation, respectively, in the xenograft tumors.

### Statistical analysis

Differences were evaluated using Student's t test, and *P* < 0.05 was considered as statistically significant. Data are expressed as the mean ± S.E.
